# Multidimensional Evaluation of the Quality of Rural Life Using Big Data from the Perspective of Common Prosperity

**DOI:** 10.3390/ijerph192114166

**Published:** 2022-10-29

**Authors:** Jing Zhang, Bingbing Huang, Xinming Chen, Congmou Zhu, Muye Gan

**Affiliations:** 1 Institute of Applied Remote Sensing and Information Technology, College of Environmental and Resource Sciences, Zhejiang University, Hangzhou 310058, China; 2 The Rural Development Academy, Zhejiang University, Hangzhou 310058, China; 3 Shandong (Linyi) Institute of Modern Agriculture, Zhejiang University, Linyi 276000, China; 4 Key Laboratory of Urban Land Resources Monitoring and Simulation, Ministry of Natural Resources, Shenzhen 518000, China; 5 Territorial Consolidation Center in Zhejiang Province, Department of Natural Resources of Zhejiang Province, Hangzhou 310007, China; 6 Department of Land Resources Management, Zhejiang Gongshang University, Hangzhou 310018, China

**Keywords:** quality of rural life (QRL), common prosperity, improvement path, big data, Lin’an District

## Abstract

Evaluating and revealing the spatial differentiations of quality of rural life (QRL) is the basis for formulating rural revitalization planning to promote rural transformation and achieve common prosperity. Taking the Lin’an District of Hangzhou city in China, an economically developed mountainous area, as an example, this study explored the connotation of QRL from the perspective of common prosperity and constructed a QRL evaluation framework involving living, employment, consumption, and leisure aspects. Then, based on multi-sourced data of 270 administrative villages as the assessment unit, we revealed the spatial patterns of QRL and proposed optimization paths to improving QRL. The results showed that (1) differences in the spatial distribution of quality of rural living, employment, consumption, and leisure of Lin’an District were significant, presenting stepped, block clustering, irregularity, and scattered patterns, respectively. (2) The overall QRL was mainly at a low level, clustered spatially, distributed in a strip pattern, and with obvious road directionality. (3) Based on the evaluation results of QRL, we divided the 270 administrative villages into six types of improvement: livability, employment, consumption, leisure, and balanced and lagged development types. This study could provide a scientific cognitive basis for the improvement of QRL and a useful reference for rural revitalization in China.

## 1. Introduction

Rural and urban development as well as their relationship are always related to the development of society and the economy [1], and have thus been widely studied and discussed [2,3]. With rapid urbanization and industrialization, the economic, spatial, and social patterns of rural areas have changed, causing a series of problems, such as land abandonment, rural poverty, shortage of public facilities, cultural emptiness, and environmental pollution [4,5,6], which significantly affect the sustainable development of rural areas and inhibits the improvement of quality of rural life (QRL). In such context, rural areas are receiving increasing attention, and scientific evaluation and improvement of QRL are its important contents [7,8].

Poverty has always been one of the greatest challenges of human society; it is endemic, chronic, and stubborn. Poverty may be interpreted as a shortage of sufficient income to obtain the necessities of life, such as food, clothing, housing, transportation, and basic services, as measured primarily through household income or expenditure [9,10]. However, the use of income-based measures of poverty has been criticized, and poverty is influenced by a combination of natural, social, and economic factors, and subsequently multidimensional theories of poverty, subjective poverty [11,12,13], have been proposed to deepen the connotation of poverty. The standard of living refers to the composite of the consumption of goods and services, measured by economic development and expressed as indicators such as gross domestic product (GDP) and per capita income or wages [14,15,16,17,18].

Quality of life was first proposed in 1958 as “the comprehensive evaluation of people’s living standards” [19]; in contrast to standard of living, quality of life emphasizes more multidimensional concepts, including life satisfaction, inner contentment and self-realization in society [10,20]. This means that standard of living reflects the material aspects of the life of the population, while quality of life more comprehensively evaluates people’s living conditions. Due to different social systems and economic development patterns, scholars have had different understandings of quality of life. For example, in the US and Canada, influenced by a free economy, scholars have paid more attention to people’s individual feelings as the basis for a subjective quality of life [21]. In Slovakia, scholars have also responded to the growing interest in quality of life as being significantly associated with the term “happiness” [22]. In other parts of Europe, on the other hand, scholars have emphasized the significance of objective indicators and focused on “people’s living conditions” [23]. Compared with these countries, quality of life research in Asia, Africa, and Latin America began relatively late and lagged behind in terms of depth and breadth of research [7]. In recent years, an increasing number of scholars have advocated for the combination of subjective and objective indicators to comprehensively reflect people’s quality of life. The Eurostat report pointed to the need to complement GDP measures and other regularly used objective measures such as employment rates with subjective measures such as people’s life satisfaction [24]. People have gradually come to a consensus on the concept of “quality of life” as a multidimensional term that assesses people’s overall well-being, including both material (income, infrastructure, etc.) and nonmaterial elements (spirit, cultural quality, etc.). Quality of rural life (QRL) is a multidimensional, integrated concept for evaluating the living states of people, which often involves economic, social, cultural, political, ecological, and other aspects, reflecting both people’s satisfaction with their living conditions and the level of rural economic development and social civilization [25,26].

In response to the necessity to address rural challenges, governments have increasingly integrated the improvement of QRL into their policy goals. For instance, the EU Rural Development Policy (2007–2013) proposed improving the quality of rural life as one of its priority objectives. The Chinese central government proposed the rural revitalization strategy in 2017 [27,28], aiming to break long-standing bottlenecks, solve the three rural issues and achieve comprehensive enhancement of QRL. Additionally, the main focus of research has shifted away from the quality of urban life toward the quality of rural life; researchers have evaluated the QRL among different regions [29], analyzed the factors that influence QRL [30,31], and analyzed the development of an indicator system and technical methods for improving QRL [8]. In terms of research indicators, scholars have borrowed from the rural revitalization indicator system [32] and the urban-rural integration development indicator system [33] and added indicators such as infrastructure and cultural revitalization in addition to traditional indicators such as population, economy, and environment. The data used in the development of this evaluation index have been primarily obtained through questionnaire surveys and statistical data; index weights have mostly been determined using the hierarchical analysis method and the entropy weight method [34]; and calculations about the quality of rural life have primarily relied on the multi-objective linear weighting method, GIS spatial analysis methods, and so on [35,36]. In studies on the influence of certain variables, scholars have used spatial autocorrelation analysis [37], geographic probes, and diagnostic models of barrier factors to investigate the factors and mechanisms that most influence QRL [38]. Early studies were limited by the difficulty of data acquisition, especially because of the lack of systematic and continuous economic and social data at the village level. With the emergence of information and communications technologies and the information economy, big data bring new opportunities for research on QRL. Scholars have utilized mobile communication data [39] and point of interest (POI) data [40,41] to identify the functions of and types of development in rural areas to explore the connection between human activities and QRL.

Overall, although the academic community has achieved a series of results regarding QRL, the research based on the multidimensional comprehensive evaluation of QRL to analyze the improvement path for weak rural areas is still insufficient, especially those that combine urban-rural integration, rural revitalization, and common prosperity goals. The research conducted on this topic has mostly relied on static data from single sources, such as statistical and planning data, lacking more precise data, which leads to evaluation only to townships or counties and insufficient research on the rural scale. The evaluation results have difficulty directly guiding rural revitalization construction practice due to the lack of accurate control of the overall QRL.

In 2021, Zhejiang Province was listed as the first pilot zone of common prosperity in China. This strategy considered quality to be the first goal of development and gave new breath to Chinese people’s pursuit of a high quality of life. How to use the opportunity of common prosperity to reverse long-term rural decline and promote the comprehensive vitalization of rural areas is becoming a top priority of China’s rural development. Therefore, this study uses big data technology and means to construct a QRL evaluation index system from the perspective of the common prosperity goal, employing multisource fusion data drawn from typical rural areas in Zhejiang Province. We estimate QRL and show how the quality of life varies across regions. Finally, using self-organizing feature map neural network technology, we classify the different improvements that have taken place in QRL and propose various further improvement paths, aiming to narrow the gap between urban and rural areas and providing useful references for the rural development and revitalization of China and the the wider developing world.

## 2. Theoretical Framework for QRL

### 2.1. Historical Trajectory of QRL in China

**Figure 1:**
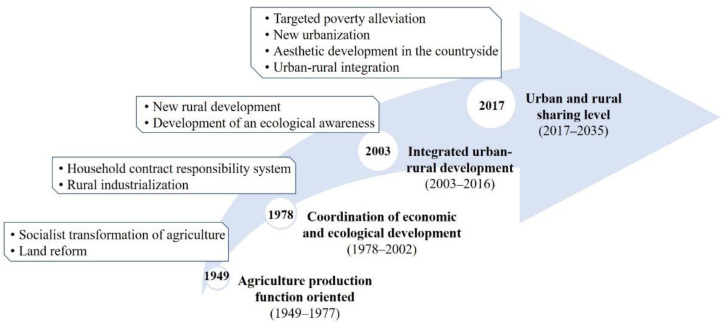
The stages characteristic of QRL in China from 1949 to 2035.

China, the largest developing country in the world, has experienced rapid urbanization. Since the founding of New China, the per capita disposable income of rural residents has increased from 49.7 yuan in 1949 to 18,931 yuan in 2021 [42]. Although the income in rural areas has increased significantly, the income gap between urban and rural areas still remains large, with an urban–rural income gap ratio of 2.50. Compared to the characteristics of China’s early economic development, developed western countries such as Europe and the US have placed great emphasis on the rural economy and the coordinated development of urban and rural areas, with policies that encourage the subsidization and development of agriculture through industry, with a small urban–rural gap [43]. Changes in the recognition of QRL over time are mainly influenced by national policies. In general, China’s rural development has gone through roughly four stages (Figure 1):

At the beginning of the founding of the People’s Republic of China, China’s rural development lagged behind, with a per capita disposable income of only 49.7 yuan for rural residents and a general lack of food and clothing, and under such pressure, the government formulated an agricultural policy oriented toward grain production [44]. The economic structure at this stage was singular, and the agriculture production function occupied the dominant position in the national economy. The priority of rural development was to stabilize the growth of grain production and achieve a basic balance between supply and demand. Therefore, QRL was mainly based on agricultural production.

In 1978, influenced by the highly centralized planned economic system and the urban–rural dual structure, production factors such as rural population, land, and capital were continuously transferred to urban non-agricultural industries. Moreover, farmland abandonment became increasingly prominent and grain production continued to decline, thus causing the deprivation of rural areas by cities and agriculture by industries. With the establishment of the household contract responsibility system, rural productive forces were liberated, rural economic development was greatly improved, and the per capita disposable income of rural residents reached 2476 yuan in 2002 [45]. At the same time, people’s demand for rural landscapes shifted from a single need to multiple needs, such as tourism development and reorganizing spatial forms. Therefore, QRL began to emphasize the coordination of economic and ecological development.

Since 2003, rural development has faced problems involving intensified contradictions between supply and demand, increased pressure on the environment, and imbalance in the allocation of urban and rural resources; thus, the income gap between urban and rural residents has continued to widen. In this context, China has promoted a new urbanization approach to strengthen the radiating effect of towns on rural development, and carried out beautiful countryside construction to improve rural conditions; the per capita disposable income of rural residents has grown at least fourfold since 2003 and the ratio of disposable income of urban and rural residents has decreased from 3.23:1 in 2003 to 2.72:1 in 2016 [46]. Agriculture is no longer the victim of modernization, and rural industrial structure and development positioning have also gradually transformed. Urban-rural factor mobility has become the focus of rural development, and QRL has gradually attracted the attention of both scholars and policymakers. In other words, QRL was reoriented toward integrated urban-rural development.

Since the 19th National Congress of the Communist Party of China (2017), the country has been working to implement a rural revitalization strategy, aiming to solve the unbalance development between urban and rural areas and inadequate development in rural areas [47]. In 2020, corresponding policy documents were proposed to redirect and guide QRL by narrowing the development gap between urban and rural areas, promoting factors to flow freely, and accelerating steps toward common prosperity. In 2021, the per capita disposable income of rural residents was 18,931 yuan, and the standards of production and living of farmers had made a big step forward. However, rural development was obviously unbalanced, the per capita disposable income of rural residents showed a decreasing trend from the coastal to western regions, with generally higher income in coastal areas such as the Yangtze River Delta and the Pearl River Delta. At this stage, QRL pays more attention to realizing urban-rural integration and improving the level of urban-rural sharing.

Overall, the focus of rural development has shifted from an initial development based on yield and aimed at food security and agricultural production to a comprehensive revitalization of rural industries, talent, culture, ecology, and organization. Moreover, the demands of rural residents have also gradually shifted from the fulfillment of material needs to the pursuit of a high quality of life.

### 2.2. QRL and Common Prosperity

In 2018, the Chinese government put up a comprehensive plan for implementing the rural revitalization strategy, which emphasized the importance of affluent life. Affluent life is represented not only by the improvement of the natural and living environment but also by spiritual and cultural aspirations. Affluent life is the fundamental purpose of rural revitalization. Improving QRL is a vital step toward increasing farmers’ sense of gain and satisfaction, as well as achieving a comprehensive upgrade of the countryside; it is also the key to “retaining people” and “raising people” in rural areas.

Rural areas have inadequate infrastructural conditions, a weak industrial base and few economic resources due to the long-term dual urban–rural economic structure. This has led to a gradual decline in QRL and also to a great loss of population in rural areas, especially the migration of middle-aged and young people to cities and regions with better development conditions, and an increasingly serious aging and hollowing out of villages. This created a vicious cycle that causes rural decline. Therefore, promoting population flow between urban and rural areas is an effective way to improve QRL.

**Figure 2:**
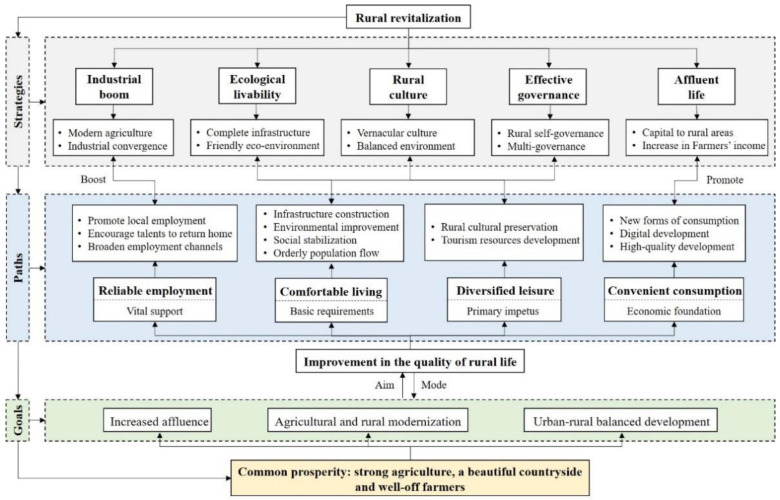
Theoretical framework of QRL.

In the 14th Five-Year Plan, China set the goal of common prosperity for all. Common prosperity has proposed a new form of civilization based on the political, economic, social, ecological, and cultural dimensions of development; it has aimed at creating a situation of overall affluence in which all people own the means of production and the means necessary to live a good life while maintaining moderate disparities between people. Rural revitalization is the necessary path to common prosperity and provides important means to achieve this prosperity; common prosperity is the ultimate goal of rural revitalization and provides the direction and impetus for rural revitalization (Figure 2).

The goal of common prosperity emphasizes narrowing the urban-rural gap, equalizing urban and rural public services and enhancing people’s ability to increase their income, which provides a new research perspective and theoretical paradigm for QRL development. The connotation of QRL is influenced by the combination of rural geographical location, production and lifestyle, socio-economic structure, institutional policies changes, and external environment. QRL is a multidimensional composite concept to measure people’s living standards and status. It is an important indicator that reflects the level of rural economic development and social civilization. It is directly related to the vital interests of rural residents and their well-being. Whether rural areas have been revitalized can be assessed through an evaluation of the quality of rural life. Therefore, accurately evaluating QRL, classifying the improvement types of QRL, and then adjusting the rural structure to improve QRL and meet people’s growing demand for a better life are the new realistic requirements for implementing the rural revitalization strategy.

### 2.3. Building an Evaluation Index System to Measure QRL

**Figure 3:**
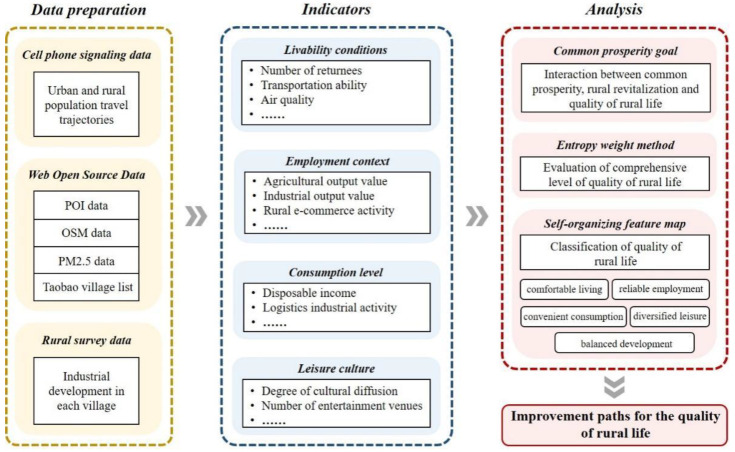
Research framework.

The improvement of QRL is essentially the process of rural transformation and internal power enhancement, and is a strategic choice for promoting the overall revitalization of rural areas. It involves the enhancement of natural environment, location conditions, and economic development. Among them, slope, elevation, hydrology, and other natural conditions are the fundamental factors that affect QRL; location conditions affect rural development opportunities, areas close to the central cities are easily driven by the city’s radiation and can better facilitate exchanges and external links between urban and rural areas; education, medical, and other public service facilities are the centralized manifestation of convenience of rural residents. Industrial development is the key to rural revitalization, accelerating the modernization of agriculture and rural areas and improving economic income are the fundamental goals of rural revitalization and the focus of QRL improvement. QRL in the new era is more concerned with urban-rural factor mobility and more equalization of public services. According to Maslow’s hierarchy of needs theory [48], the improvement of QRL can be explained as the process of changing the environment to satisfy the growing needs of the residents under the influence of the external environment and the increasing income of people (Figure 3).

QRL should include suitable living quality, convenient transportation quality, harmonious natural quality, good employment quality, consumption quality, and leisure and cultural quality. Referring to the evaluation index system of rural revitalization and urban–rural integration constructed by scholars [48,49,50], we comprehensively considered the basic principles of scientific, systematic and representative of index selection, as well as the directivity and data accessibility of QRL evaluation. In this study, the evaluation index system of QRL in Lin’an District was constructed from four aspects, including livability conditions, employment context, consumption level, and leisure culture (Table 1).

Livability conditions represent the basic daily needs of residents and are an important factor in the quality of rural life [51]. In the past, this aspect was influenced by factors in the natural environment, such as topography and landscape; however, in recent years, the effects of socioeconomic factors, such as socioeconomic development and transportation networks, have intensified. Today, livability conditions are reflected in indicators such as the number of returnees, transportation capacity, the number of hospitals and schools, and air quality [52]. The employment context has guaranteed rural residents’ participation in economic development. Indeed, a favorable employment context is greatly significant to improving QRL. Such improvement is characterized by the total output value of the rural agricultural and industrial economy and how much people benefit from the integration of rural agriculture with rural e-commerce activities [53]. Furthermore, consumption constitutes the economic basis for QRL. Increased consumption among rural residents has broken through the constraints of the traditional business model, and a new rural consumption system has emerged, as demonstrated by rural residents’ per capita disposable income and the logistics activities and number of stores in rural areas [54]. The culture of leisure has been a crucial development in rural areas; it has provided people with intellectual support and spiritual impetus for rural development, which manifests itself in two primary ways: cultural dissemination and cultural experience. These two aspects of development are expressed through cultural diffusion, the number of entertainment venues in rural areas, and rural tourism development [55].

## 3. Materials and Methods

### 3.1. Study Area

**Figure 4:**
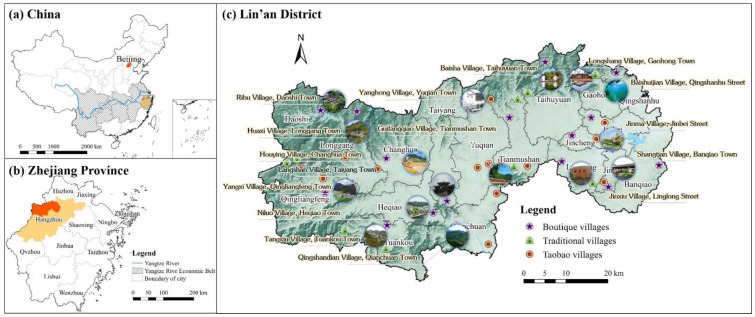
The study area of Lin’an District. (**a**) The location of Lin’an District in China. (**b**) The location of Lin’an District in Zhejiang Province. (**c**) Distribution of typical villages in Lin’an District.

Zhejiang Province, located at the intersection of the “Belt and Road Initiative” area and the Yangtze River Economic Belt, has been deemed a pilot zone for quality of life improvements and the implementation of the common prosperity campaign (Figure 4). Lin’an District has been strategically modernizing and urbanizing and was the first locality to integrate major national, provincial, and municipal schemes to accelerate the overall development of its regional urban nodes and foster its competitive advantage. The district has provided one of the first batches of digital village pilot areas in the nation and has focused on the development of digital agriculture display areas. It features a strong rural base, with 30 scenic villages, 18 provincial boutique villages, 14 traditional villages, 4 clusters of rural online entrepreneurs (Taobao towns), and 19 smaller Taobao villages. A scenic village is defined as a village oriented toward rural tourism, introducing professional operation teams, revitalizing landscape resources, and forming a certain market scale of agricultural tourism and cultural tourism integration development. A boutique village is defined as an upgraded specialty village with obvious natural environmental or humanistic characteristics, having greater potential in forming local competitive brands and promoting farmers’ income. A Taobao village is defined by the Ali Research Institute as a village with at least 10% of its residents operating online stores and annual sales of at least 10 million yuan. Taobao town refers to the existence of 3 or more than Taobao villages or annual e-commerce sales exceeding 30 million yuan of a town [56].

In recent years, the economy has grown rapidly, which has contributed to individuals increasingly demanding better living conditions in rural areas. This development has greatly modified the allocation of resources in rural areas and the spatial structure and systematic function of Lin’an District; it has also affected the quality of development within rural areas toward high-quality development. Therefore, this paper selects the Lin’an District of Hangzhou as its study area because it is representative of and provides insight into new ways to revitalize rural areas and achieve collective prosperity.

### 3.2. Data Source and Processing

Based on the available data and the timeliness of the research, this study focused on 2021 as its time frame and included Lin’an District’s 270 administrative villages as its scope. The data included cell phone signaling data, open-source web data and rural survey data.

Cell phone signaling data were collected by an urban and rural development research team at the Zhejiang Mobile Data Center. Through the analysis of mobile travel track data, it was possible to obtain data such as the monthly population flow from towns to villages and the monthly number of short-term tourists in each administrative village in the study area. The open-source web data mostly included POIs, information about roads, and a list of Taobao villages. POI data were acquired from the application programming interface provided by Baidu Map Open Platform Web Service, which mainly referred to geographic entities with spatial identification and attributed information related to rural life. Road data were obtained from Open Street Map. Rural survey data mainly contained the area’s per capita disposable income and the total value of industrial production in each village. Socioeconomic data on population, infrastructure, public services, etc., were drawn from the 2021 *Lin’an Statistical Yearbook* and *Zhejiang Statistical Yearbook*.

### 3.3. Methods

#### 3.3.1. Data Standardization

To eliminate the dimensional impacts of different evaluation indicators and make the results comparable, the indicators were standardized using the extremum standardized method [57]. If a greater value was more favorable to the development of the system, then the index was positive, and Equation (1) was used for its standardization. If a lower value was more favorable to the development of the system, then the index was negative, and Equation (2) was used for its standardization. All the index values were mapped to [0, 1]. The calculation formulas were as follows:

Positive index:

(1) $$y_{ij}=\frac{x_{ij}-x_{\min}}{x_{\max}-x_{\min}},(i=1,2,\cdots ,m;j=1,2,\cdots n),$$

Negative index:

(2) $$y_{ij}=\frac{x_{\max}-x_{ij}}{x_{\max}-x_{\min}},(i=1,2,\cdots ,m;j=1,2,\cdots n),$$

where $y_{ij}$ denotes the standard values for raw data and $x_{ij}$ denotes the specific index value of the j evaluation indicator for rural areas i.

#### 3.3.2. Indicator Weight Set

The entropy weight method is an objective weighting method that avoids bias from subjective influence to a certain extent and has been widely used in the evaluation of environmental science, urban planning and rural revitalization. It mainly determines the weight according to the amount of information transmitted by each index. The larger the entropy value of an index is, the less information is transferred, that is, the lower the weight of the index is [58]. This paper used the entropy weight method to determine the weight of each evaluation index. The specific steps were as follows.

The information entropy of the index was determined:

(3) $$E_{j}=-\left(\frac{1}{\ln z}\right)\sum_{i=1}^{z}p_{ij}\ln\left(p_{ij}\right),$$

(4) $$p_{ij}=\frac{y_{ij}}{\sum_{i=1}^{z}y_{ij}},$$

where $E_{j}$ is the information entropy of the *j*-th evaluation index, and z is the number of evaluation units (z = 270 in this paper).

The index weight was determined:

(5) $$W_{j}=\frac{1-E_{j}}{\sum_{i=1}^{m}\left(1-E_{j}\right)},$$

where $W_{j}$ is the weight of the *j*-th evaluation index, and m is the number of evaluation indexes (m = 15 in this paper).

#### 3.3.3. Evaluation Model for the Quality of Rural Life

Combined with the standardized value and weight of each evaluation index, the comprehensive QRL in each evaluation unit was calculated. The calculation formula was as follows:

(6) $$G_{i}=\sum_{i=1}^{m}y_{ij}w_{j},$$

where $G_{i}$ is the degree of the comprehensive development level of rural areas, $G_{i}\in \left[0,1\right]$.

#### 3.3.4. Self-Organizing Feature Map

The self-organizing feature map (SOFM) is an unsupervised artificial neural network model that is self-adaptive, self-organized, and self-learning [59]. It can automatically learn from or simulate its surroundings to adjust its own network structure. The SOFM network, which maps high-dimensional data into a low-dimensional space, can extract the internal rules of complex distribution patterns by bringing similar sample grids closer to each other on the output plane [60]. That is, input data with similar features are gathered together, and conversely, data with different features are scattered. This method has been widely applied to classification studies in ecology and land use planning [61,62].

In this study, to explore spatial combination regularities in QRL, we identified quality of life bundles at the village level using the Python third-party libraries’ minisom classification function. The training epochs were limited to 270; the classification number was set to 3 and then 8; and the other parameters were set to the default settings. The four indicators of living condition, employment context, consumption level, and leisure culture were dimensionless processed by range standardization and constructed into a 6 × 270 training matrix, which was input into the SOFM model for training, and the partition result was obtained.

## 4. Results

### 4.1. Characteristics of Each Dimension of QRL

To scientifically understand the difference in QRL, we classified the rural livability, employment, consumption and leisure culture of Lin’an District into four levels of quality of life: low, relatively low, relatively high and high through the mean partition method. There was significant spatial variation in QRL in Lin’an District, and QRL was good in the east and worse in the west.

**Figure 5:**
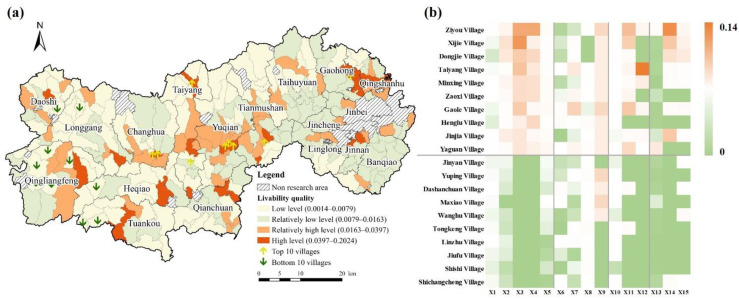
Performance of livability quality in Lin’an District. (**a**) Livability conditions index. (**b**) Top 10 villages and bottom 10 villages. The top 10 villages are above the horizontal line; the bottom 10 villages are below the horizontal line; X1–X5 represent livability condition indicators; X6–X9 represent employment context indicators; X10–X12 represent consumption level indicators; and X13–X15 represent leisure culture indicators.

The quality of living was highest, with evaluation values ranging from 0.0014 to 0.2024 and an average value of 0.0163. The high-value areas were mainly located along the G56 Hangrui Expressway, with a stepped down trend along the expressway alignment to the northwest, southeast, and southwest (Figure 5). With the radiation effect of superior transportation conditions, the rural areas along the road have relatively well-equipped infrastructure, close urban–rural interactions, diversified life of residents, and a more suitable living environment. The villages in the top 10 have certain advantages in transportation, education, and medical resources. In contrast, the areas where people experienced a low quality of life accounted for a larger proportion, indicating that the rural population of Lin’an District lived with overall little residential comfort. The low-value areas were distributed in the western region because of its mountainous concentration, severely limited transportation, distance from central cities, and lack of infrastructure construction.

**Figure 6:**
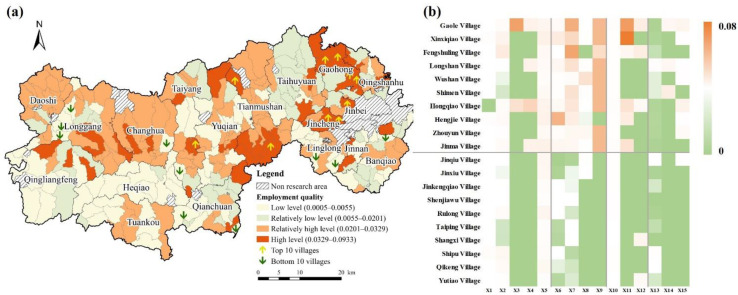
Performance of employment quality in Lin’an District. (**a**) Employment context index. (**b**) Top 10 villages and bottom 10 villages.

The quality of employment was the lowest. The evaluation values ranged from 0.0005 to 0.0933, with an average value of 0.0201 (Figure 6). The relatively high-value and high-value areas were block clustered, mostly situated in the northern part of the Nanshao River and Tianmu River. The northeastern Lin’an District is the demonstration area of high-standard farmland. The main industries are rice planting and commercial vegetable and potato production. It has a long history of agricultural production, strong competitiveness in agricultural modernization, and an excellent agricultural infrastructural and economic foundations. The top 10 villages mainly benefited from the deep integration and development of the agricultural industry and e-commerce. The relatively low-value and low-value areas were mainly located in the southwest. The primary cause was that the areas were heavily influenced by topography, the agricultural inputs were relatively low, the development of the secondary industries was relatively poor, and the level of the agriculture-based economic development was relatively insufficient.

**Figure 7:**
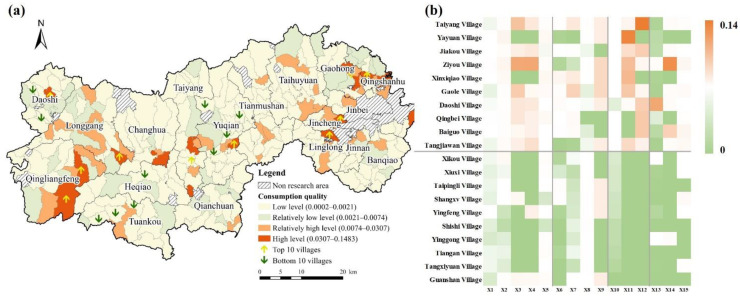
Performance of consumption quality in Lin’an District. (**a**) Consumption level index. (**b**) Top 10 villages and bottom 10 villages.

The quality of consumption was low, with evaluation values ranging from 0.0002 to 0.1483 and the average value of 0.0074. There was a significant regional differentiation in the spatial distribution of consumption quality (Figure 7). Areas where consumption was high and relatively high were irregularly distributed and narrowly covered, with a total proportion of only 18.51%, and these areas were scattered in the northeast and southwest, mainly on Qingshanhu Street. Due to the attraction and radiation of the Hangzhou West Science and Innovation Corridor, the northeast part of Lin’an District has a high level of economic development, convenient transportation, close urban–rural exchanges, diverse sources of livelihood for villagers, and high per capita income. The top 10 villages not only had certain advantages in the performance of consumption quality but also performed better in terms of livability and employment quality. The areas where consumption was relatively low and low were characterized by a concentrated contiguous distribution throughout the district, indicating that overall consumption in Lin’an District was low.

**Figure 8:**
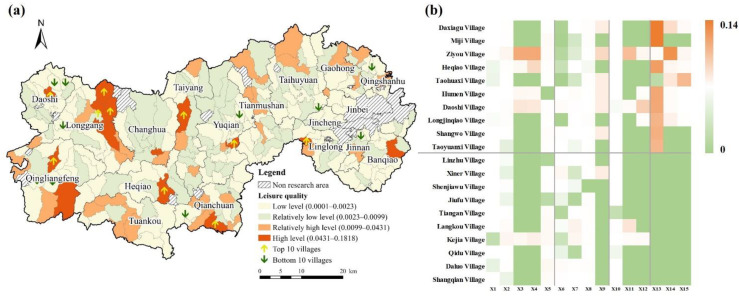
Performance of leisure quality in Lin’an District. (**a**) Leisure culture index. (**b**) Top 10 villages and bottom 10 villages.

The quality of leisure was high, with evaluation values ranging from 0.0001 to 0.1818 and an average value of 0.0099 (Figure 8). The relatively high-value and high-value areas were mainly located in the southwestern section and the mountainous hilly areas, with a more scattered pattern. The topographic features have a significant impact on distribution characteristics. The western part of Lin’an District has high vegetation coverage, a relatively high topographic relief degree and better ecological resources. These regions were located far from the core urban development area, were less disturbed by human activities, and had a relatively sound ecological environment, which indicated that only a few villages could assume a significant leisure function. The top 10 villages usually had worse performance in livability, employment, and consumption quality. The low value areas were mainly distributed in blocks across the eastern region, covering the widest area and accounting for 51.11% of the total area. This is mainly because these areas were flat and rural settlements and their residents had greater artificial disturbance to the natural world.

### 4.2. Comprehensive Characteristics of QRL

**Figure 9:**
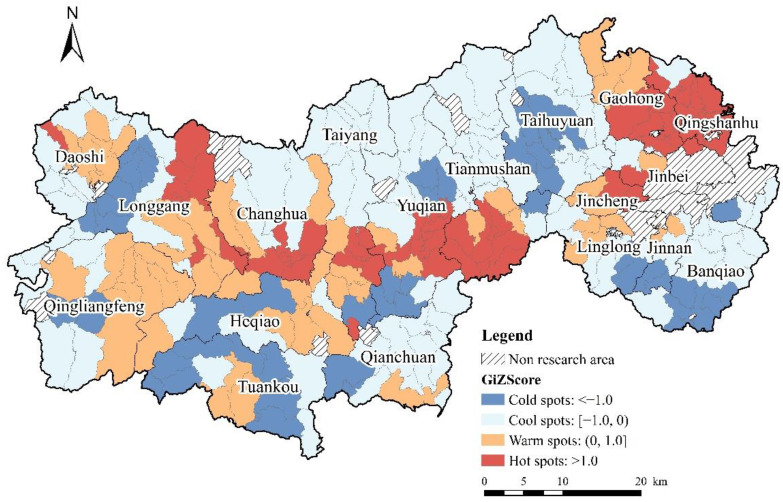
Distribution of hot spots of QRL in Lin’an District.

Based on the evaluation of quality of living, employment, consumption, and leisure, we derived the combined value of QRL level of administrative villages in Lin’an District using the weighted summation method (Figure 9). The combined value of QRL level of Lin’an District was mainly low, and the average value was 0.0537. There were 87 administrative villages with QRL values greater than the average, accounting for 32.2% of all villages. The Getis–Ord G* index was used to identify hot spots and cold spots of QRL to reflect the spatial heterogeneity of rural development in Lin’an District (Figure 9). Through a global spatial clustering check (Getis–Ord General G) and spatial “hot spot” detection visualization, we found that high-value areas of QRL were concentrated in three large hot spots, mainly in the valley plain areas along the G56 Hangrui Expressway, in a “strip” distribution. In particular, they were most concentrated in Qingshanhu Street, Tianmushan and Changhua Town. These regions have the relative advantages of superior location, equipped commercial services, and economic conditions, which support rural high-quality development. Low-value QRL areas were concentrated in four large cold spots, mostly in areas with steep mountains and narrow valleys, and poor accessibility of rural roads, leading to inconvenience in production and life, and limiting the improvement of the overall QRL level. The other areas had no obvious spatial agglomeration characteristics. In general, rural areas with flat terrain, well-developed transportation, public service facilities and sound industrial foundation had better overall QRL, and vice versa.

### 4.3. Type of Improvements in QRL

**Figure 10:**
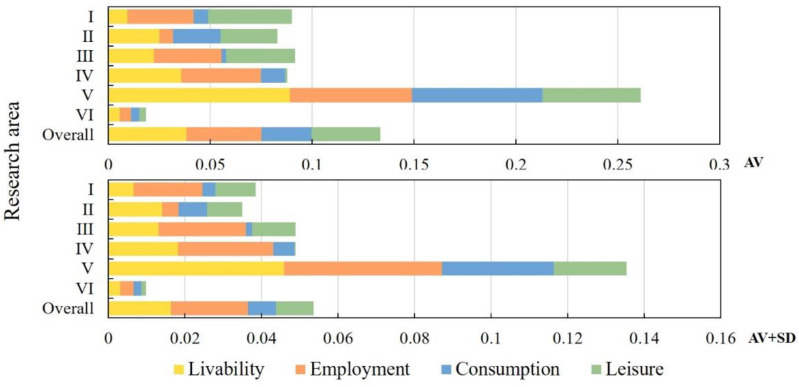
Basic characteristics of different types of enhancement areas.

Based on the spatial characteristics of QRL, and using Yang’s division strategy [54], we considered that when the sum of the mean and standard deviation of a certain quality of life index in the area was less than the sum of the mean and standard deviation of the quality of life index in the overall study area, we could identify that the quality of life represented an improvement in QRL; when the development area did not need to improve the quality of life, it was identified as a balanced development area (Figure 10). Based on this classification, areas I, II, III, IV, V, and VI were identified as the main areas for progress in livability, employment, consumption, and leisure, as well as the balanced and lagged development areas.

**Figure 11:**
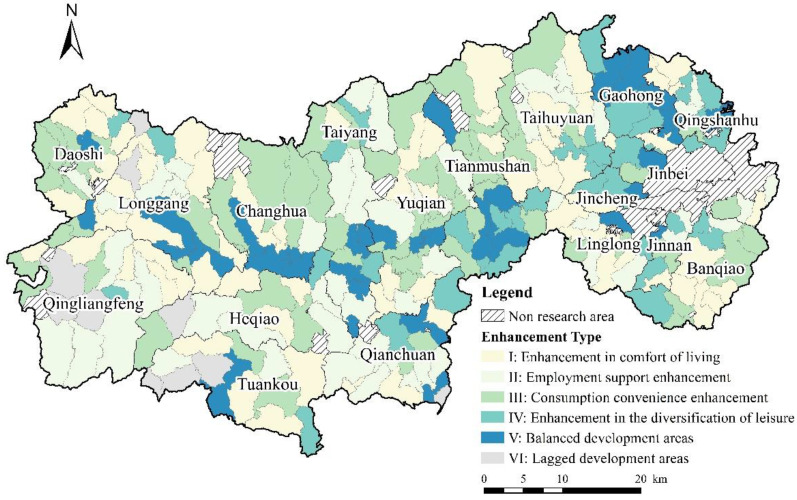
Distribution of enhancement types of QRL.

Figure 11 shows that the regions of the lagged development type are mainly distributed in western Lin’an District, including 15 villages in Xinjiang, Hebei, and other towns, accounting for 33.35%, which indicated that the unbalanced and insufficient rural development in Lin’an District is still serious. The balanced development type was the most ideal state for QRL, but this kind of village was relatively few and was scattered along the G56 Hangrui Expressway, accounting for 13.33%. These regions had a developed economy and were highly urbanized. Benefiting from the radiating drive of cities and spillover effects, the rural industrial structure had undergone a significant transformation and upgrading, and had created conditions for rural economic development and farmers’ employment.

There were relatively more livability-oriented improvement types, mostly located in mountainous and hilly areas or traditional agricultural villages, including 78 villages, indicating that the undulating terrain and poor location conditions may cause increased difficulty in infrastructure construction and reduce the sharing effect of urban and rural service resources, resulting in weaker livability and less comfort in this area. Employment-oriented improvement types were scattered in the southwest, where the terrain was relatively steep and not suitable for large-scale agricultural production. This has caused great inconvenience to people’s production and life and has limited the development of agriculture and secondary industries. The consumption-oriented improvement type was the second largest, mainly located north of the G56 Hangrui Expressway. The villages in these areas were lagging behind in economic development and were highly dependent on agriculture compared to neighboring areas, with a more homogeneous industrial model and limited income sources for farmers, resulting in lower QRL. Leisure-oriented improvement types were mostly distributed in the northeast. These regions have a developed economy, and the regional natural condition is poor, indicating that rural economic development may have a negative impact on the ecological environment. This hinders the further development of villages.

## 5. Discussion

### 5.1. An Analytical Framework Based on “Common Prosperity”

In the earlier stage of rural development, natural geographical environment was considered to be a fundamental constraint to the enhancement of QRL [25]. Elevation and slope are important indicators reflecting the topography of the rural areas, which are the basic natural conditions affecting agricultural production and infrastructure construction and have an essential impact on the differentiation of QRL. Areas with low-value QRL in the villages generally have high slopes and altitudes, poor access to rural roads, low penetration of education and medical services, and inconvenient living conditions for residents. After entering the ages of industrialization and urbanization, people began to create material wealth continuously, and the impact of different regions’ economic development levels and economic structures on QRL became more and more prominent [46]. A shift from a single agriculture to a multifunctional industry has been achieved in the rural areas, with a steady improvement in the rural landscape and a continuous increase in farmers’ income. The clustering distribution of QRL advantageous areas is highly similar to the spatial distribution of the development of economic level. Rural areas with a high proportion of secondary and tertiary industry output value and frequent flow of various factors between urban and rural areas have increased income of residents and relatively high QRL. As can be seen, traditional natural resource conditions continue to play a vital role, but the economic impact on QRL is becoming increasingly evident. With the in-depth promotion of new rural construction, precise poverty alleviation and other strategies, residents’ requirements for living standards are no longer limited to meeting basic material needs, but begin to pursue a higher quality of life. At this stage, the principal contradiction facing Chinese society is between the people’s ever-growing needs for a better life and unbalanced and inadequate development. QRL is more focused on the development of urban and rural integration. Therefore, the evaluation of QRL should be considered from various aspects, focus on the sharing and exchange of urban and rural resources, and keep in mind the needs of different residents for a better life. In other words, QRL is a concept with rich content and scientific connotations, and is a dynamic process of gradual progress in line with the laws of economic and social development. A basic consensus has been that QRL comprises two aspects: material life, which includes living conditions and residents’ income; and spiritual life, which considers residents’ personal feelings and lifestyles. The process from low quality and basic development to high quality and innovative development has been dynamic and continuous. Accordingly, people have stopped being satisfied with only their basic needs being fulfilled and instead have begun to pursue a better quality of life.

This study constructed an analytical framework based on livability conditions, employment context, consumption level and leisure culture, emphasizing a common prosperity orientation. This is mainly reflected in the orderly mobility of the population, the equalization of infrastructure public services between urban and rural areas, and the increase in farmers’ income. Constructing an evaluation index system for QRL based on common prosperity goals was a novel approach that revealed the overall degree to which rural areas have developed. The results were helpful in providing guidelines for improving the quality of life in urban and rural areas, promoting the development of urban and rural integration, formulating policies, and further realizing the equivalence between urban and rural areas.

### 5.2. Spatial Characteristics of QRL Based on Big Data

Evaluating, monitoring, and improving QRL have played an important role in supporting rural revitalization and achieving common prosperity. To date, evaluation methods have mainly entailed isolated research projects based on statistical data or questionnaire surveys, and comprehensive evaluations combining subjective and objective methods have not been extensively developed. This paper thus introduced big data. Through big data, we efficiently obtained diverse data pertaining to the study area. The use of these multisource data compensated for the shortage in traditional data, especially due to a lack of access to various types of data at the village scale, and these multisource data also improved the timeliness and scientificity of our evaluation. The evaluation index system was more dynamic, specifically including the number of returnees, the activity of rural e-commerce, and the frequency of logistics industrial activities, focusing on the two-way flow between urban and rural areas.

The results show that the overall QRL was high in the east and low in the west of Lin’an District. The average quality of employment was the highest; the quality of livability was the second highest; the quality of leisure was weaker; and the quality of consumption was the lowest. These results were probably related to Lin’an District seizing the historical development opportunity of “Turning counties into districts”, steadily developing agriculture, accelerating the integration of industry and city, and hastening the transformation of the “Two Mountains” while having insufficient and imbalanced development in its rural areas. This phenomenon has been gradually emerging in some rural areas in China, such as Beijing and Shanghai [63,64].

### 5.3. QRL Improvement Paths

Scientifically classifying the different ways in which QRL can improve is a key step in the promotion of rural development and rural revitalization. With the characteristics of QRL in Lin’an District, this paper used the SOFM neural network on the Python platform to classify the rural areas into six improvement types (Figure 7):

Livability optimization type: The livability of this type of village was low. The infrastructure and public service facilities were poor. However, the population flow was high. The nonagricultural characteristics of residents were significant. The government should provide basic welfare and economic assistance to ensure living, medical care, education and other services for the villagers [52]. In addition, the government should provide vocational training and employment opportunities to enhance the development capabilities of villagers. This can promote the sustainable development of rural areas.

Industrial transformation type: This mainly included employment-oriented improvement villages. The rural livability of this type of village was good, which can basically meet the living and development needs of farmers. However, its industry output was low. The government should create a specific industrial chain based on local characteristic industries to realize industrial scale operations and provide more employment opportunities for farmers. This can attract and retain people [65,66]. Characteristic crops should be cultivated and developed, and characteristic high-efficiency agriculture and agricultural product processing industries should be developed in hilly areas. This can promote the scale, industrialization, and standardization of agriculture.

Consumption transformation type: Opportunity for strategic digital reform should be seized in Hangzhou, and the comprehensive integration of modern digital technology in rural areas should accelerate, which will narrow the “digital divide” between urban and rural areas [67]. Second, a specialty e-commerce platform in Lin’an District should be created, and the scale of online marketing of agricultural products such as pecans, bamboo shoots, and tea should be expanded to reach efficiency in agricultural output and increase farmer’s income.

Cultural tourism optimization type: This mainly included leisured-oriented improvement villages. The government should first combine the region’s ecological resources with the mountainous characteristics of Lin’an District, develop rural tourism, and take advantage of ecological resources for economic gains [68]. Second, Lin’an’s regional characteristics and culture should be used, literary and artistic works created, and Lin’an’s cultural brand and competitiveness gradually improved.

Demonstration type: This type of village had the highest QRL, showing a belt-like layout along the expressway, with significant location advantages. It had well-developed public service facilities and infrastructure and outstanding resource advantages. Residents had a high degree of employment, mainly relied on nonagricultural income and have a high economic level. The priority should first be given to the development of pilot villages in these regions as a demonstration effect. This practice should then be promoted in other areas [58]. Second, security mechanisms, such as in planning and construction management, should be explored to ensure long-term rural development.

Layout optimization type: This was mainly concerned with rural areas that are lagging behind in development. These types of villages were geographically closed, bound by topographic conditions, with low industrial efficiency and inadequate infrastructure support, and were clearly disadvantaged in various aspects compared to the surrounding areas. This seriously hinders the further development of villages. Based on the wished of local residents, we should actively lead and arrange residents to live in areas with relatively superior natural environment, service facility conditions and industrial foundation through relocation forms. The government should proactively improve the conditions of connectivity in the vast rural areas, promote the modernization of infrastructure and public service facilities in rural areas, and enhance the convenience of residents’ lives and the efficiency of social resource services.

### 5.4. Limitations and Further Research

Evaluating the quality of rural life using big data has been useful. This study was innovative in that it established a more systematic measure of QRL under the Common Prosperity campaign. The study also introduced big data to obtain village-scale data and used the SOFM neural network to cluster-analyze the quality of rural life in each village. Our evaluation process and conclusions are helpful to policymakers. However, this study chose only one year to study QRL levels without longitudinal comparisons across time, which makes it more challenging to describe the progression process of QRL levels and rural development. Future research should focus on the evolutionary process and shaping mechanisms. To encourage related work, the following considerations are proposed: with access to data acquisition channels, the degree of rural flow could be measured for different factors from the perspective of that flow [30], and key factors affecting QRL could be identified. The interaction between characteristics and spatial differences among factors was analyzed, and changes in regional organization and spatial patterns were revealed. Finally, the ways in which rural characteristics have flowed and become concentrated were explored to test the evaluation effect of the index system.

## 6. Conclusions

QRL reflects the overall characteristics of rural development, as well as the level of satisfaction of the residents with their living conditions. Improving QRL is key in the future of rural revitalization and a basic prerequisite for achieving common prosperity. This study established an analytical framework and indicator system to evaluate QRL in Lin’an District and provided an effective quantitative evaluation using village-level big data. The following conclusions were drawn.

The QRL of Lin’an District presented obvious spatial variations. The quality of living showed a stepwise decline towards the northwest, southeast and southwest with the major traffic roads as the horizontal axis. The quality of employment was high in the central and eastern regions and low in the western region, showing obvious regional differences. The quality of consumption was comparatively low, and the distribution was not centralized, where the high-value areas were irregular and scattered across the north of the G56 Hangrui Expressway. The high-quality leisure area was staggered in the southwest, while the low value was located in the northeast, following a “cluster” pattern. The overall level of QRL was relatively low, and its spatial distribution had significant horizontal agglomeration. The overall level of QRL showed a trend of decreasing radiation from the main traffic arteries to the southwestern, southeastern and northwestern directions, having a clear traffic orientation. With the characteristics of QRL, Lin’an District was classified into six improvement types: livability optimization type, industrial transformation type, consumption transformation type, cultural tourism optimization type, demonstration type, layout optimization type. Next, this study proposed improvement paths suitable for each type, which can provide a reference for village planning and QRL improvement in similar areas.

With the continuous differentiation and integration of various elements within rural areas, the focus of QRL also continues to develop and evolve. This study summarized the stage characteristics of the evolution of rural development since the founding of the People’s Republic of China. The spatial pattern of QRL was described from a quantitative perspective, and the types of each region were divided based on the classification method of weak quality. The research results were helpful in enriching the connotation and extension of QRL, further understanding the fit between the current construction and actual residents’ needs, and laying a foundation for improving the weak quality of rural areas in each county. The path of QRL improvement is complex and diversified, involving a wide range of aspects, including changes in policy orientation, social and economic development, cultural landscape upgrading and consumer demand, and transition of the relationship between urban and rural areas. China’s specific national conditions compound the uncertainty and complexity of the improvement of QRL, its successful implementation requires consideration of all aspects. People are the main part of the rural revitalization, while developing and guiding the improvement of QRL, we should also fully consider the loss of rural population, guard against the decline of traditional industries and culture and the pollution of the village’s natural environment, so as to realize the sustainable development of rural areas.

Achieving common prosperity is the ultimate goal of rural revitalization, and whether the rural areas are revitalized can be measured by the level of QRL. Evaluating and improving QRL is a complicated social practice, and long-term follow-up research on the dominant constraints in rural areas should be pursued in the future to deepen and widen the connotation of QRL, thus advancing rural development and ultimately making agriculture stronger, villages more beautiful, and farmers richer.

## Figures and Tables

**Table 1:** Evaluation index system for QRL in Lin’an District and weights of indexes.

Target	Indicators	Explanation	Weight
Livability conditions	Number of returnees	The change value of rural household population at the end and the beginning	0.003
	Transportation ability	Including road class and length	0.031
	Number of hospitals	Number of hospitals per 10,000 people in rural areas	0.109
	Number of schools	Number of schools per 10,000 people in rural areas	0.092
	Air quality	Average monthly air quality compliance days	0.009
Employment context	Total output value of rural agricultural economy	The general situation of rural agricultural output	0.044
	Total output value of rural industrial economy	The general situation of rural industrial output	0.052
	Benefits of rural agricultural integration	The level of integration of primary, secondary and tertiary industries in the countryside and the level of benefit distribution	0.024
	Rural e-commerce activity	Number of e-commerce platforms	0.041
Consumption level	Economic income of rural residents	Per capita disposable income of rural residents	0.003
	Frequency of logistics industrial activities	Number of trips made by delivery riders	0.126
	Number of stores	Number of stores per 10,000 people in rural areas	0.135
Leisure culture	Degree of cultural diffusion	Search volume by search engines for matching keywords	0.138
	Number of entertainment venues	Number of entertainment venues per 10,000 people in rural areas	0.117
	Degree of development of rural leisure tourism	Number of agritourism establishments and homestays run by rural residents	0.075

## Data Availability

Not applicable.
